# Pancreatic Steatosis Associates With Impaired Insulin Secretion in Genetically Predisposed Individuals

**DOI:** 10.1210/clinem/dgaa435

**Published:** 2020-07-29

**Authors:** Róbert Wagner, Benjamin Assad Jaghutriz, Felicia Gerst, Morgana Barroso Oquendo, Jürgen Machann, Fritz Schick, Markus W Löffler, Silvio Nadalin, Falko Fend, Alfred Königsrainer, Andreas Peter, Dorothea Siegel-Axel, Susanne Ullrich, Hans-Ulrich Häring, Andreas Fritsche, Martin Heni

**Affiliations:** 1 Institute for Diabetes Research and Metabolic Diseases of the Helmholtz Center Munich at the University of Tübingen, Tübingen, Germany; 2 German Center for Diabetes Research (DZD e.V.); 3 Department of Internal Medicine, Division of Diabetology, Endocrinology and Nephrology, University Hospital Tübingen, Tübingen, Germany; 4 Section on Experimental Radiology, University Hospital Tübingen, Tübingen, Germany; 5 Institute of Pathology and Neuropathology, University Hospital Tübingen, Tübingen, Germany; 6 Department of General, Visceral and Transplant Surgery, University Hospital Tübingen, Tübingen, Germany; 7 Interfaculty Institute for Cell Biology, Department of Immunology, University of Tübingen, Tübingen, Germany; 8 Department of Clinical Pharmacology, University Hospital Tübingen, Tübingen, Germany; 9 Institute for Clinical Chemistry and Pathobiochemistry, Department for Diagnostic Laboratory Medicine, University Hospital Tübingen, Tübingen, Germany

**Keywords:** beta cell function, insulin secretion, non-alcoholic fatty pancreas disease, pancreatic steatosis, prediabetes, type 2 diabetes

## Abstract

**Context:**

Pancreatic steatosis leading to beta-cell failure might be involved in type 2 diabetes (T2D) pathogenesis.

**Objective:**

We hypothesized that the genetic background modulates the effect of pancreatic fat on beta-cell function and investigated genotype × pancreatic fat interactions on insulin secretion.

**Design:**

Two observational studies.

**Setting:**

University hospital.

**Patients or participants:**

A total of 360 nondiabetic individuals with elevated risk for T2D (Tuebingen Family Study [TUEF]), and 64 patients undergoing pancreatectomy (Pancreas Biobank [PB], HbA1c <9%, no insulin therapy).

**Main Outcome Measures:**

Insulin secretion calculated from 5-point oral glucose tolerance test (TUEF) and fasting blood collection before surgery (PB). A genome-wide polygenic score for T2D was computed from 484,788 genotyped variants. The interaction of magnetic resonance imaging-measured and histologically quantified pancreatic fat with the polygenic score was investigated. Partitioned risk scores using genome-wide significant variants were also computed to gain insight into potential mechanisms.

**Results:**

Pancreatic steatosis interacted with genome-wide polygenic score on insulin secretion (*P* = 0.003), which was similar in the replication cohort with histological measurements (*P* = 0.03). There was a negative association between pancreatic fat and insulin secretion in participants with high genetic risk, whereas individuals with low genetic risk showed a positive correlation between pancreatic fat and insulin secretion. Consistent interactions were found with insulin resistance-specific and a liver/lipid-specific polygenic scores.

**Conclusions:**

The associations suggest that pancreatic steatosis only impairs beta-cell function in subjects at high genetic risk for diabetes. Genetically determined insulin resistance specifically renders pancreatic fat deleterious for insulin secretion.

Pancreatic steatosis (ie, pancreatic fat) is characterized by an increased pancreatic triacylglycerol content and embodies adipocytes interspersed in the pancreatic parenchyma (1). Accumulating evidence implicates this fat compartment as a modulator of islet function (1-4). Specifically, pancreatic steatosis was inversely associated with insulin secretion in subjects with prediabetes (2). Further, it has been suggested that reduction of pancreatic steatosis is pivotal in improving beta-cell function during a lifestyle intervention (5, 6). However, subjects with normal glucose tolerance do not exhibit the negative association between pancreatic steatosis and insulin secretion (2, 7). In longitudinal studies, there was no association between age-adjusted computed tomography-measured pancreatic steatosis and diabetes incidence (8), but only in lean subjects (9). In patients with type 2 diabetes (T2D), pancreatic fat associated with a reduction of glucagon-stimulated insulin secretion (10). A possible explanation to these controversial findings could be a large heterogeneity of individual responses to increased pancreatic fat mass. As a complex disease, T2D results from an interplay of genetic predisposition and environmental factors (11). We hypothesized that unfavorable effects of pancreatic steatosis on beta-cell function arise only in the context of a genetic predisposition for diabetes.

## Methods

### Subjects

In the discovery analyses, we investigated data from 360 nondiabetic subjects of the Tübingen Family Study (TUEF) who underwent magnetic resonance imaging (MRI) measurements of pancreatic fat content (see Supplementary Figure 1a which is located in a digital research material repository (12)). The subjects were recruited based on their elevated risk for type 2 diabetes (positive family history, prior gestational diabetes, known glucose intolerance, or overweight) and underwent oral glucose tolerance tests (OGTT). Subjects either had normal glucose tolerance or impaired fasting glucose and/or impaired glucose tolerance. Subject characteristics are given in Supplementary Table 1. All supplementary material and figures are located in a digital research materials repository (12). Liver fat measurements were missing in 10 subjects, C-peptide-based insulin secretion measurements were missing in 12 subjects. In the validation analysis, we investigated data (N = 64) from our Pancreas Biobank. Patients undergoing pancreatic surgery provided written informed consent to donate pancreas tissue for research purposes. We obtained macroscopically healthy tissue that had been resected during surgery, but was not needed for further pathology workup. Fasting blood was drawn before surgery to provide detailed metabolic phenotyping. Patients on insulin therapy and those who had very high HbA1c levels independent from therapy (>9%) were excluded from the analysis (Supplementary Figure 1b, located in a digital research materials repository (12)). Patient characteristics are shown in Supplementary Table 2, located in a digital research materials repository (12).

### Measurement of insulin secretion

Participants of the TUEF study underwent 5-point OGTT with 75 g glucose. Blood samples were taken at fasting and after 30, 60, 90, and 120 minutes.

Insulin secretion was measured from stimulated insulin and C-peptide levels using the insulinogenic index (13) and area under the curve (AUC)-C-peptide_0-30_/AUC-glucose_0-30_. The AUCs for C-peptide and glucose were calculated with the trapezoid method. Insulin sensitivity was assessed by the insulin sensitivity index of Matsuda and DeFronzo (14). Participants of the Pancreas Biobank provided fasting blood samples before surgery. In this cohort, insulin secretion and sensitivity were computed using the homeostatic model assessment 2 (HOMA2) method (15). Insulin secretion was computed from fasting glucose and C-peptide (HOMA2%B) or fasting glucose and insulin for a sensitivity analysis (HOMA2%B-insulin). Insulin sensitivity was computed from fasting glucose and insulin levels (HOMA2%S).

Plasma glucose was measured using a bedside glucose analyzer (glucose-oxidase method, Yellow Springs Instruments, Yellow Springs, OH) or from sodium fluoride plasma using an automated glucose oxidase method on Siemens ADVIA XPT Clinical Chemistry Analyzer (Siemens, Healthineers, Erlangen, Germany). Plasma insulin and C-peptide were measured using the ADVIA Centaur XPT Analyzer (Siemens Healthineers, Eschborn, Germany). HbA1c measurements were performed using the Tosoh glycohemoglobin analyzer HLC-723G8 (Tosoh Bioscience, Tokyo, Japan).

### Quantification of pancreas fat, liver fat, and other adipose tissue compartments

**Figure 1.**
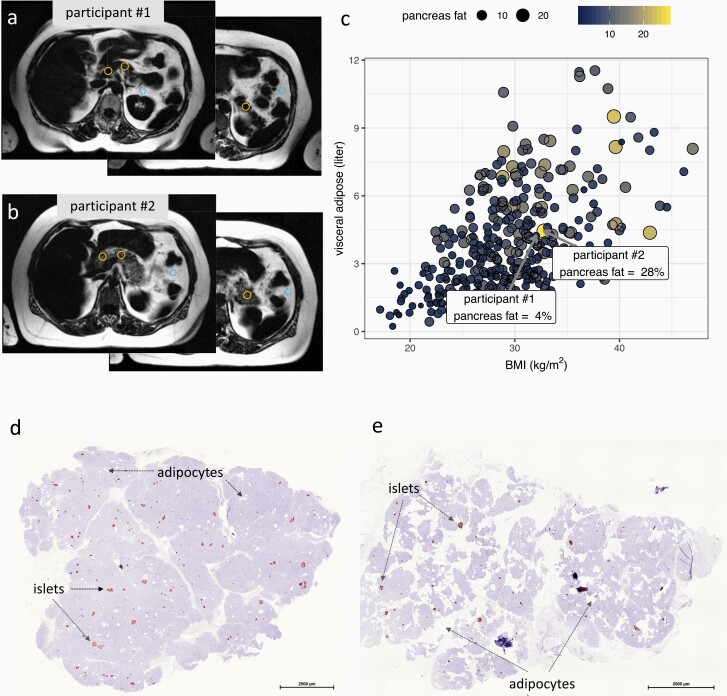
Determination of pancreatic fat content. In the discovery cohort, pancreatic fat was measured from cross-sectional fat selective magnetic resonance imaging (MRI) images using the average value of the regions of interest at the head, neck, and tail of the organ (orange circles; a, b). Nearby visceral adipose tissue (dotted blue circle) is used as internal reference. MRI scans (a, b) are from 2 female study participants. Participant 1 (46 years, BMI 32 kg/m^2^) has a pancreatic fat content of only 4% (A), whereas participant 2 (66 years, BMI 33 kg/ m^2^) has 28% (B). Participants with similar BMI and visceral adipose tissue can have strikingly different pancreatic fat contents (C; n = 360, dot color and size indicate pancreatic fat content). In the validation cohort, pancreatic fat was determined from hematoxylin and eosin-stained tissue slices also featuring insulin costaining to locate islets of Langerhans (D, E). One patient (D) had low pancreatic fat content, whereas the other (E) high pancreatic fat content.

In the discovery cohort, pancreatic steatosis was quantified by MRI as previously described (Fig. 1A, B) (16). MRI is considered as the most accurate method to quantify pancreatic steatosis (16). The evaluation of the MRI scans was performed by a single experienced investigator (J.M.), who was not aware of the subject’s characteristics. The regions of interest (ROIs) of the pancreas were focused on the caput, corpus, and cauda. The mean of the ROIs was calculated for each individual. The ROIs of pancreatic fat were measured avoiding macroscopic vessels, motion artefacts, or partial volume artefacts. As internal reference ROI of the adjacent mesenteric fat was used. This reference was close to the investigated pancreatic tissue. Even participants with similar body mass index (BMI) and visceral adipose content displayed a considerable heterogeneity in pancreatic fat content (Fig. 1C). Liver fat content, which was used as a covariate in some of the models, had been measured by localized proton magnetic resonance spectroscopy, as described previously (17).

In the validation cohort (N = 64), pancreatic steatosis was assessed from insulin immunostained histological sections with a manual and an automatic method (Fig. 1D, E). First, fat infiltration levels were manually specified as none (N = 9), < 10% (N = 25), and > 10% (N = 30) by an experienced operator (F.G.) who was unaware of the laboratory results and the genetic risk score at the time of the investigation. For further analyses, the 2 lower categories were aggregated, resulting in a low (N = 34) and high (N = 30) pancreatic fat infiltration group. Second, we used an automatic segmentation of adipocyte area on histological sections of each patient using ImageJ (18, 19).

Pancreatic tissue samples were obtained from the tumor-free resection margins, as provided by the pathologist. Paraffin-embedded pancreatic sections were incubated with primary antibody against insulin (1:1000; Dako) and CD68 (1:3000, Cell Signaling Technology). The primary antibody was detected using the Opti-View system (Ventana, Multimer Technology, Roche). Hematoxylin-eosin was used as counterstaining and for the assessment of the grade of fibrosis. Macrophage/monocyte infiltration was quantified by counting the CD68^+^ cells in islets.

Visceral adipose tissue volume was quantified between femoral heads and thoracic diaphragm from axial T1-weighted fast spin-echo MRI as described in Machann et al (20) by an automatic segmentation procedure based on fuzzy clustering and orthonormal snakes (21). Liver fat content was assessed by single-voxel proton MR spectroscopy applying a stimulated echo acquisition matrix technique with short echo time (10 ms) and long repetition time (4 seconds). Ratio of the integral of methyl/methylene and water signal was calculated and liver fat content is given as percentage of the entire signal (20).

### Genotyping and polygenic scores

We used multiple approaches to estimate genetic diabetes risk. An outline on polygenic risk scores is provided in the review of Udler et al (22). First, we computed a genome-wide polygenic score (gwPS), also known as global extended polygenic score, for each participant using the combination of all genotyped variants and BMI-adjusted summary statistics from the latest large genome-wide association study meta-analysis in ~900,000 European subjects (23). This computation was performed with the LDPRED algorithm, which aggregates Bayesian estimates for each variant after accounting for linkage disequilibrium (24). After quality control, exclusion of multiallelic and low-frequency variants, we combined 484,788 variants from the datasets, yielding an estimated genome-wide single-nucleotide protein (SNP)-heritability of 0.069 in the TUEF cohort. Correlation of this polygenic risk score with glycemic endpoints in the whole TUEF cohort is shown in Supplementary Table 3 (12). Second, we computed polygenic scores from a restricted set of genome-wide significant variants (restricted to significant polygenic scores) that were partitioned to pathomechanistic groups. Specifically, we assigned variants to homeostatic action groups (insulin sensitivity, insulin secretion, or both) using data from Mahajan et al (25). The provided glycemic traits of interest were HOMA-IR and insulin sensitivity index-Matsuda (for insulin sensitivity), as well as HOMA-B and corrected insulin response (CIR; for insulin secretion). We selected all genome-wide significant variants for diabetes that were affecting 1 or more of these specific glycemic traits at *P* values < 0.025 resulting in the classification of SNPs shown in the Supplementary Table 4 (12). Because effect sizes for different outcomes (HOMA-indices, CIR, etc.) were difficult to consolidate into meaningful weights, scores were computed by simple addition of risk alleles. Third, we used the partitioning of variants to different pathomechanistic clusters as suggested by Udler et al (26). In brief, this work assigned variants to one or more of the following groups: (1) variants affecting beta-cell function with elevated proinsulin (beta-cell); (2) beta-cell function with reduced proinsulin (proinsulin); (3) obesity; (4) lipodystrophy; and (5) liver/lipid. All genotyping in the TUEF cohort and in the participants of the pancreas biobank was performed using a 700-K Infinium Global Screening Array from Illumina (San Diego, CA).

### Statistics

All statistical analyses were conducted in R version 3.4 (27). For linear regression models, outcome variables were log-transformed to approximate normal distributions. To facilitate comparison of genetic effects for different outcomes, effect sizes are shown as standardized estimates (β), with outcomes normalized to a mean of 0 with standard deviations of 1. In the statistical models, insulin secretion was always adjusted for insulin sensitivity (Matsuda-index or HOMA%S). In the models using restricted-to-significant polygenic scores partitioned to mechanism of action, we adjusted insulin secretion (insulinogenic index) to insulin sensitivity, sex, age, age^2^, BMI, and visceral adipose tissue volume. A *P* value of < 0.05 was considered statistically significant.

### Study approval

Both studies conformed to the principles outlined in the Declaration of Helsinki and the study protocols had been approved by the institutional review board at the Medical Faculty of the University of Tübingen. Written informed consent from all participants was received before inclusion in the study. For the study, all participants are identified only by number, not by name.

## Results

### Genome-wide polygenic score using MRI measured pancreatic steatosis

Genetic T2D risk (gwPS) inversely associated with insulin secretion (AUC-C-peptide_0-30_/AUC-glucose_0-30_) after adjustment for insulin sensitivity, sex, age, age^2^, and BMI (β = -0.038, *P* = 0.019). However, there was no association between gwPS and pancreatic steatosis in the discovery cohort (*P* = 0.21). Pancreatic steatosis also did not associate with insulin secretion when analyzed in the full cohort (after adjustment for insulin sensitivity, sex, age, age^2^, and BMI; *P* = 0.166 for AUC-C-peptide_0-30_/AUC-glucose_0-30_). Correlation of pancreatic fat content with anthropometric, glycemic, and lipid variables is shown in Supplementary Table 5 (12).

In models testing interaction of pancreatic steatosis with gwPS, the interaction term was significant for the insulin secretion indices (β = -0.097, *P* = 0.008 for insulinogenic index; and β = -0.95, *P* = 0.011 for AUC-C-peptide_0-30_/AUC-glucose_0-30,_ adjusted as described previously). These interactions were also independent from the potential confounders visceral adipose tissue and liver fat content (*P* = 0.006 and *P* = 0.001 for the interaction terms in the insulin- and C-peptide-based secretion assessment, respectively, full multivariable models are shown in Supplementary Tables 6–7 (12)).

**Figure 2.**
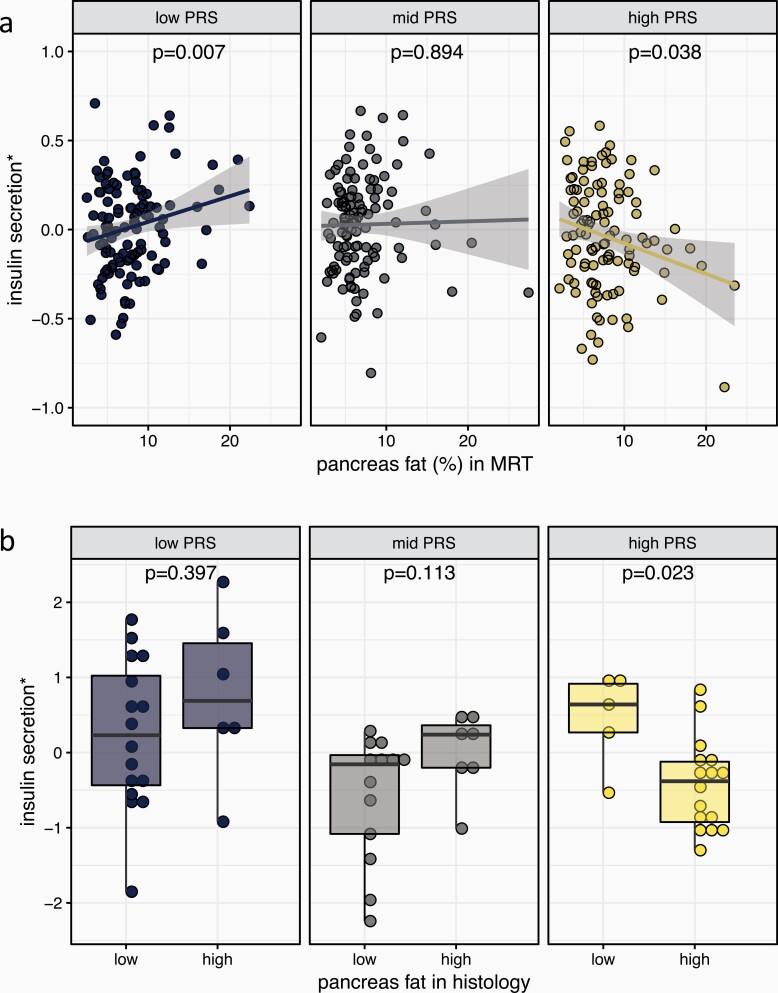
Association of pancreatic fat content with insulin secretion depends on genetic risk to develop type 2 diabetes. Strata of polygenic risk scores (PRS, determined as genome-wide polygenic scores) are shown in 3 panels with different colors for low, middle, and high PRS. (A) In the discovery set, pancreatic fat was measured by MRI. Insulin secretion was determined from OGTT and adjusted for confounders (y-axis: standardized residuals of log-transformed AUC-C-peptide_0-30_/AUC-glucose_0-30_ adjusted for sex, age, age^2^, BMI, insulin sensitivity (Matsuda index), visceral adipose tissue, and liver fat). (B) In the validation set, pancreatic fat was estimated from immunohistological sections as shown in Fig. 1. Insulin secretion was determined from the HOMA2%B index (y-axis: standardized residuals adjusted for insulin sensitivity (HOMA2%S), sex, age, and BMI). *P* values are from linear regression models. AUC, area under the curve; BMI, body mass index; HOMA2, homeostatic model assessment 2; MRI, magnetic resonance imaging; OGTT, oral glucose tolerance test.

When stratifying the study population by terciles of genetic risk (gwPS low, mid, and high), pancreatic steatosis positively associated with insulin secretion (AUC-C-peptide_0-30_/AUC-glucose_0-30_) in the low gwPS group (β = 0.091, *P* = 0.007) and negatively in the high gwPS group (β = -0.086, *P* = 0.038; Fig. 2A).

### Genome-wide polygenic score using histologically determined pancreatic steatosis

When analyzing the validation cohort as a whole, there was no association of genetic risk or pancreatic steatosis with insulin secretion (*P* > 0.3). However, we detected an interaction between gwPS and pancreatic fat infiltration (*P* = 0.03 with the dichotomized manual pancreatic fat measurements and *P* = 0.0001 using mean adipocyte coverage area from automatically quantified fat measurements) on C-peptide-based insulin secretion. The interaction was also significant for insulin-based HOMA2-B index (*P* = 0.03 and *P* = 0.02, respectively). In the low and mid gwPS strata, pancreatic fat did not associate with insulin secretion (*P* > 0.112). In the high gwPS stratum, participants with high histologically determined pancreatic fat infiltration had lower insulin secretion (β = -0.880, *P* = 0.011, Fig. 2B). Dichotomized data from automatic measurements of pancreatic fat showed a directionally consistent trend for association (Supplementary Figure 3 (12)). Patients with higher pancreatic fat content in their histologic sections had also larger insulin staining areas, but no differences in the gwPS (Supplementary Figure 4 (12)). We did not detect an association of the degree of pancreatic steatosis with underlying pancreatic disease, obstruction of the pancreatic duct, or histologic grade of tissue fibrosis (Supplementary Table 8 (12)).

### Restricted to significant polygenic scores partitioned to mechanism of action

In a further step, we computed polygenic scores only comprising genetic variants that are associated with T2D at a genome-wide significant level. These genetic variants were grouped to broad categories of mechanism of action. In the first realization of this approach, we used published data of effects on glycemic traits such as insulin sensitivity indices and insulin secretion indices to assign the variants to specific categories. From these scores, only the group of variants affecting insulin sensitivity was involved in the interaction with pancreatic fat (Table 1). When examining the interaction of clustered genetic scores with pancreatic fat content, we identified an interaction for the group of SNPs involved in the liver/lipid phenotype suggested by Udler et al (26) (Tables 1 and 2).

**Table 1.** Interaction Between Pancreatic Fat Content and Partitioned Diabetes Risk Scores on Insulin Secretion Grouped by Action on Metabolic Homeostasis

Score Type	Effect Estimate	SE	*P* Value
Secretion	-0.019	0.041	0.6
Secretion/sensitivity	-0.026	0.03	0.4
Sensitivity	-0.093	0.031	0.003

Insulin secretion is measured by the insulinogenic index. Effect estimates, standard errors and *P* values are provided for the interaction term (risk score × pancreatic fat content) in models additionally adjusted for insulin sensitivity, age, age^2^, sex, body mass index, visceral adipose tissue volume). Risk scores use the sum of diabetes risk alleles in variants grouped by action on metabolic homeostasis (modulating insulin secretion, insulin sensitivity or both).

**Table 2.** Interaction Between Pancreatic Fat Content and Diabetes SNP-Clusters on Insulin Secretion

SNP-Cluster	Effect Estimate	SE	*P* Value
Beta-cell	0.04	0.056	0.5
Lipodystrophy	0.005	0.053	0.9
Liver/lipid	-0.116	0.056	0.04
Obesity	0.029	0.053	0.6
Proinsulin	-0.057	0.051	0.3

Insulin secretion is measured by the insulinogenic index. Effect estimates, standard errors and *P*-values are provided for the interaction term (risk score × pancreatic fat content) in models additionally adjusted for insulin sensitivity, age, age^2^, sex, body mass index, visceral adipose tissue volume). SNP-clusters are computed as described by Udler et al (26).

Abbreviation: SNP, single-nucleotide polymorphism.

## Discussion

Our data provide evidence that pancreatic fat has distinct association patterns with insulin secretion depending on genetic T2D risk. In case of low genetically determined T2D risk, there was a positive association of pancreatic steatosis with insulin secretion in the discovery cohort. In contrast, subjects with a high genetic T2D risk showed lower insulin secretion levels associated with increased pancreatic steatosis. Because these associations were independent of BMI, insulin sensitivity index, MRI-measured visceral adipose tissue, and liver fat content—all bona fide confounders of insulin resistance—the impact of pancreatic steatosis on insulin secretion appears to be independent from whole-body insulin action but highly dependent on genetic predilection to diabetes. The lower insulin secretion in case of high pancreatic fat content and elevated genetic risk was validated in a different set of patients with histologically quantified pancreatic fat content.

Data from the partitioned polygenic scores provide insights on the biological background of the observed interaction. We identify an interaction between the insulin-sensitivity modulating group of SNPs and pancreatic fat content, suggesting that variants determining insulin resistance are involved in flipping the action of pancreatic fat on insulin secretion to negative. Among the polygenic scores proposed by Udler et al (26), the liver/lipid cluster showed a consistently directed interaction with pancreatic fat (ie, higher polygenic risk led to a more negative slope of the association of pancreatic fat with insulin secretion).

These findings complement recent experimental data from human pancreatic adipocytes and islets cocultures showing that pancreatic fat cells lead to a deterioration of islet function only in a typical environment that characterizes insulin resistance (28). Specifically, the hepatokine fetuin-A, which is typically increased in fatty liver disease, induced the secretion of inflammatory cytokines from preadipocytes and adipocytes in the presence of palmitate (28). Of note, palmitate is secreted as part of very low density lipoproteins (VLDL) from the liver. This process is amplified in persons with insulin resistance resulting from enhanced hepatic de novo lipogenesis (29). The existence of a metabolic crosstalk between pancreatic fat, fatty liver, and pancreatic beta-cells is now supported by the interaction between the liver/lipid polygenic risk cluster and the amount of pancreatic fat on insulin secretion. Of note, capturing complex relationships that underly such a multiorgan crosstalk in human subjects is challenging. The individual metabolic environment shows considerable within-person or day-to-day variation and characteristic biomarkers are not fully known or difficult to measure. Thus, genetic instruments can be used as substitutes either for the general predisposition to develop T2D (gwPS), or for specific features of the pathology (partitioned polygenic scores).

The gene × environment interaction between gwPS and pancreatic steatosis leads to a turning of the direction of association between pancreatic steatosis and insulin secretion, dependent on the genetic background (Fig. 2A).

When analyzed in an unstratified cohort, opposing association directions can wipe out each other. This may explain the lack of association of pancreatic steatosis with insulin secretion and diabetes reported in some clinical studies (7, 8, 30).

The elevated insulin secretion with higher pancreatic fat content, seen in low-risk individuals in our data, warrants further examination. Whether this phenomenon is secondary to insulinotropic effects of free fatty acids and could play a role in a perpetuation of insulin hypersecretion especially in individuals with pancreatic steatosis are important questions that currently cannot be answered from our data.

Our work shows the genetic-risk dependent association of pancreatic fat in a diabetes-prone population using gold standard measurements for pancreatic fat. Although the findings were replicated in a different cohort with complementary methods, the data in the validation cohort are limited by restricted regions of sampling, available amount of tissue, and potential collateral effects of pancreatic diseases underlying the surgery.

The findings show that pancreatic fat is relevant in the decrease of insulin secretion. However, this factor has to be interpreted in the context of genetically determined insulin resistance. Further studies have to examine the metabolic effects of pancreatic steatosis in relation to genetic risk for diabetes and other potential effect-modifying factors.

## Data Availability

The datasets generated during and/or analyzed during the current study are not publicly available but are available from the corresponding author on reasonable request.

## Abbreviations

AUC: area under the curve
BMI: body mass index
CIR: corrected insulin response
gwPS: genome-wide polygenic score
HOMA2: homeostatic model assessment 2
MRI: magnetic resonance imaging
OGTT: oral glucose tolerance test
ROI: region of interest
SNP: single-nucleotide polymorphism
T2D: type 2 diabetes
TUEF: Tuebingen Family Study

## Additional Information

***Disclosure Summary:*** The authors have nothing to disclose.
